# Development of Polyelectrolyte Complex Nanoparticles-PECNs Loaded with Ampicillin by Means of Polyelectrolyte Complexation and Ultra-High Pressure Homogenization (UHPH)

**DOI:** 10.3390/polym12051168

**Published:** 2020-05-20

**Authors:** Nicolle Montero, Maria J. Alhajj, Mariana Sierra, Jose Oñate-Garzon, Cristhian J. Yarce, Constain H. Salamanca

**Affiliations:** 1 Laboratorio de Diseño y Formulación de Productos Químicos y Derivados, Departamento de Ciencias Farmacéuticas, Facultad de Ciencias Naturales, Universidad ICESI, Calle 18 No. 122-135, Cali 760035, Colombia; nicollemontero76@gmail.com (N.M.); mariajoalhajj@hotmail.com (M.J.A.); marisierraa29@gmail.com (M.S.); cjyarce@icesi.edu.co (C.J.Y.); 2 Grupo de Investigación en Química y Biotecnología (QUIBIO), Facultad de Ciencias Básicas, Universidad Santiago de Cali, calle 5 No. 62-00, Cali 760035, Colombia; jose.onate00@usc.edu.co

**Keywords:** ampicillin, antimicrobial activity, bottom-up and top-down method, polyelectrolyte complex nanoparticles-PECNs, ultrahigh-pressure homogenization-UHPH

## Abstract

This study was focused on synthesizing, characterizing and evaluating the biological potential of Polyelectrolyte Complex Nanoparticles (PECNs) loaded with the antibiotic ampicillin. For this, the PECNs were produced initially by polyelectrolytic complexation (bottom-up method) and subsequently subjected to ultra-high pressure homogenization-UHPH (top-down method). The synthetic polymeric materials corresponding to the sodium salt of poly(maleic acid-*alt*-octadecene) (PAM-18Na) and the chloride salt of Eudragit E-100 (EuCl) were used, where the order of polyelectrolyte complexation, the polyelectrolyte ratio and the UHPH conditions on the PECNs features were evaluated. Likewise, PECNs were physicochemically characterized through particle size, polydispersity index, zeta potential, pH and encapsulation efficiency, whereas the antimicrobial effect was evaluated by means of the broth microdilution method employing ampicillin sensitive and resistant *S. aureus* strains. The results showed that the classical method of polyelectrolyte complexation (bottom-up) led to obtain polymeric complexes with large particle size and high polydispersity, where the 1:1 ratio between the titrant and receptor polyelectrolyte was the most critical condition. In contrast, the UHPH technique (top-down method) proved high performance to produce uniform polymeric complexes on the nanometric scale (particle size < 200 nm and PDI < 0.3). Finally, it was found there was a moderate increase in antimicrobial activity when ampicillin was loaded into the PECNs.

## 1. Introduction

In the last decade, there has been a remarkable increase in research related to nanoparticles (NPs) applied to the medical field, ranging from inorganic to organic systems [1,2,3,4,5,6,7,8,9,10,11,12,13,14,15]. In the case of inorganic NPs, the systems usually reported correspond to metallic nanoparticles (silver, gold, zinc oxide, iron oxide, among others) [16,17,18,19,20,21,22,23], which can be classified into three groups (I) Metallic Nanostructures (II) Metallic Oxide Nanostructures and (III) Metallic Nanoalloys, which can be produced by chemical, physical and biosynthetic methods [24]. On the other hand, the organic NPs describe a wide variety, where dendrimers [25,26], self-assembling systems (micelles and liposomes) [27,28,29,30] and polymeric nanoparticles are the most used and reported systems [31,32]. Regarding polymeric NPs, these are commonly employed in the biomedical field due to their characteristics of compatibility, biodegradability and low cytotoxicity [24], where natural polymers like chitosan, modified starches and natural gums are very usual [33]. However, the development of polymeric NPs is not exclusive to biopolymeric systems, since they can also be obtained with biocompatible synthetic materials, which are mainly homopolymers and copolymers of polylactide (PLA) [34,35], polycaprolactone (PCL) [36], polyglycolic acid (PGA) [37,38] and acrylate-based polymers [39,40,41,42,43]. Likewise, the polymeric NPs are subclassified according to their architecture, where the most usual correspond to nanospheres [44,45,46,47,48], nanocapsules [8,49,50,51,52], polymer nanoconjugates [53,54,55,56,57], and polyelectrolyte complexes (PECNs) [58,59,60,61,62]. Regarding PECNs, these can be obtained by several methodologies, being the traditional process of polyelectrolytic complexation (bottom-up method) one of the most used [63,64,65,66,67]. This technique is based on the spontaneous interpolymeric aggregation given by the electrostatic attraction, when a cationic and anionic polyelectrolyte are mixed in aqueous solution. This polyelectrolytic complexation seems in principle to be an easy methodology to perform; however, it is the opposite, since it depends on multiple intrinsic and extrinsic variables [66]. Some of these intrinsic variables are the chemical nature, molecular weight, and fraction charge of the polymer. In contrast, extrinsic variables depend on several dissolution medium conditions (temperature, pH, ionic strength, i.a.), as well as many process conditions such as polyelectrolyte ratio, mixture volume, rate and order of addition and stirring speed during the polyelectrolyte complexation. These factors lead to the fact that polyelectrolyte complexes are difficult to obtain, especially on a nanometric scale [68]. Thus, the standardization and scale-up of PECNs obtained through bottom-up methodologies is a great challenge, which involves carrying out arduous experimentation. On the other hand, there are other procedures (top-down methods) that can also be employed to obtain systems at nanometric scale. Some of these are the sonication and the homogenization by high (HPH) and ultra-high pressure (UHPH), which lead to the disaggregation of matter by the application of high energy [69,70,71]. Concerning UHPH, this is a relatively new technique and it has been used mostly in the food sector and specifically in the dairy sector, describing very interesting results [72,73,74]. This technique is based on a high energy dispersion process, in which a coarse system is passed through a micrometer diameter nozzle, causing high turbulence, shear and cavitation [75]. However, these top-down methods also have some disadvantages, where chemical degradation of the material by an excess of energy applied during the disaggregation process is one of these [72]. Nevertheless, the great benefit of this technique is that the implemented conditions can be easily reproduced and scaled at an industrial level and therefore, the NPs developed under these methodologies are suitable for an easy technology transfer. In this way, one of the main problems generated during polyelectrolytic complexation is the obtention of systems with large particle size and high polydispersity, which could be improved if it is complemented by other methods such as UHPH. 

To date, many cationic and anionic polyelectrolytes have been described and reported for the development of this type of system. However, the complexes formed between the polymeric salts derived from Eudragit-E100™ (EuCl) and sodium salt of poly(maleic acid-*alt*-octadecene) (PAM-18Na) have not yet been studied. Such combination of polyelectrolytes is very interesting because both polymers have previously demonstrated a capability to form polymeric nanocomplexes with the ampicillin antibiotic [40,76]. Therefore, the first objective of this study is focused on developing a new system of PECNs based on two known polymeric materials (EuCl and PAM-18Na), but they have not yet been combined. The second objective is focused on demonstrating how the UHPH technique may be a very interesting complementary tool for the development of PECNs, improving several of the usual problems generated during polyelectrolytic complexation (bottom-up method). The third and last objective is the evaluation of the antimicrobial effect of such PECNs loaded with a model antibiotic (ampicillin) on sensitive and resistant *S. aureus* strains.

## 2. Materials and Methods

### 2.1. Materials

The polymeric salts of PAM-18Na (monomeric unit: 412 g/mol, M_n_:30–50 kDa, ionization degree: 99%) and EuCl (monomeric unit: 435 g/mol, M_n_: 47 kDa, ionization degree: 38%) were provided by the Laboratory of Design and Formulation of Chemical Products from Icesi University (Cali, Colombia) and they were used in the form that they were received. Likewise, all the data on the synthesis and characterization of these materials were previously reported [77,78]. Tecnoquimicas S.A. Pharmaceutical Company (Cali, Colombia) provided the ampicillin. *Staphylococcus aureus* ATCC25923, ATCC29213, and ATCC43300 were purchased from Microbiologics Inc.^©^ (St. Cloud, MI, USA) and were reconstituted according to the supplier guidelines. The nanoparticle suspension was mixed with ultra-pure water that was obtained from a purification system (Millipore Elix Essential, Merck KGraA, Darmstadt, Germany).

### 2.2. Preparation of Ampicillin-Loaded Polyelectrolyte Complex Nanoparticles

**Figure 1:**
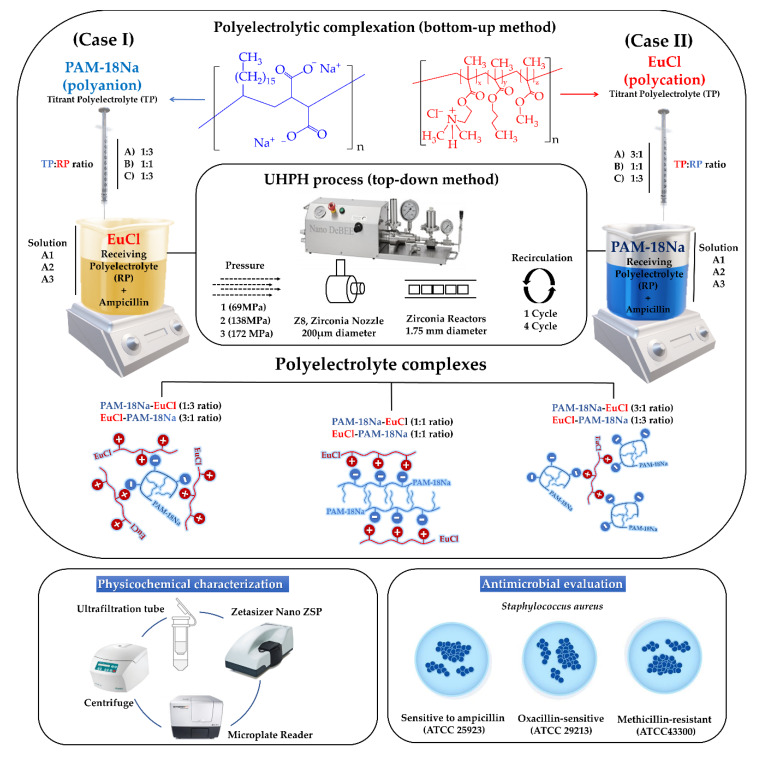
Scheme of the elaboration process, physicochemical characterization, and antimicrobial evaluation of ampicillin-loaded polyelectrolyte complex.

The schematization of the elaboration process and the physicochemical characterization of the polyelectrolyte complexes, as well as the experimental conditions employed, are depicted and summarized in Figure 1 and Table 1, respectively. The complexes obtained initially by polyelectrolytic complexation (bottom-up method) are referred as the control. In contrast, those polyelectrolyte complexes subjected to UHPH (top-down method) are named as PECs in a general way or as PECNs, only when they reach a particle size < 200 nm. 

The development of PECs was carried out in several stages according to the order of the titrant and receiving polymers, proportion of the titrant polymer, and UHPH applied pressure and recirculation cycles. Initially, two polymeric solutions of PAM-18Na and EuCl were independently prepared in ultra-pure water at a concentration of 1 mg/mL. The PAM-18Na solution was assisted by sonication (energy intensity of 1878 W corresponding to an amplitude of 60% and a pulse of 5 s followed by 10 s of rest for a total time of 18 min). Subsequently, each polymeric solution was used as a titrant or receiving system depending on the situation. For case I, PAM-18Na and EuCl were utilized as titrant polyelectrolyte (TP) and receiver polyelectrolyte (RP), respectively, whereas EuCl and PAM-18Na were used as TP and RP for case II. Additionally, different ampicillin amounts (40, 60, and 120 mg) were solubilized in 25 mL of each receiving polyelectrolyte solution, forming the solutions A1, A2, and A3, respectively. Then, different volumes of titrant polymer solution (8.33, 25 and 75 mL) at a concentration of 1 mg/mL were added to each respective solution A (~300 rpm, 25 °C) to form the suspensions of polyelectrolytic complexes (PECs). The polyelectrolytic complexation was carried out using different molar proportions according to each respective monomeric unit (1:3, 1:1, and 3:1), leaving the ampicillin at a fixed concentration of 1.2 mg/mL at the end of the complexation. Once the ampicillin-loaded PECs were formed, the suspensions were subjected to UHPH using a parallel flow configuration, zirconium nozzle (Z8, diameter: 200 µm), and six zirconium reactors (diameter 0.95 mm). Likewise, different operational conditions that corresponded to the number of cycles (1 and 4) and pressure (68.9, 137.9 and 172.4 MPa) were varied and in this stage, the PECNs are formed.

### 2.3. Physicochemical Characterization of Nanoparticles

Particle size, polydispersity index (PDI), and zeta potential analyses were determined using a Zetasizer nano ZSP (Malvern Instrument, Worcestershire, United Kingdom) that was equipped with a red He/Ne laser (633 nm). Particle size and PDI were measured using dynamic light scattering (DLS) [79] with a scattered angle of 173° at 20 °C and a quartz flow cell (ZEN0023), whereas the zeta potential was measured using a disposable folded capillary cell (DTS1070). DLS measures the diffusion of particles submitted to Brownian motion and use a correlation function on the instrument that corresponds to the Stokes–Einstein relationship, which allows us to obtain the particle size and size distribution parameters. Such correlation function is acquired by a cumulative analysis and is fitted to a simple exponential, where the mean size (z-average diameter) and an estimate of the distribution width (polydispersity index) are obtained. Likewise, the correlation function also fits a multiple exponential, where the particle size distribution is acquired as non-negative least squares (NNLS) or constrained regularization (CONTIN). In this work, we report the particle size as the z-average diameter, and the PDI ranges from 0 to 1, corresponding to a monodisperse and very wide distribution, respectively. All the nanoparticles were dispersed in ultra-pure water employing a ~1:100 v/v dilution factor. All measurements were performed in triplicate and reported as the mean ± standard deviation.

### 2.4. Encapsulation Efficiency (EE)

The *EE* of ampicillin was assessed using the ultrafiltration/centrifugation technique. An aliquot of each polyelectrolyte complex suspension was transferred into an ultrafiltration tube (VWR, modified polyethersulfone-PES 10 kDa, 500 µL, diameter: 0.96 cm) and centrifuged (MIKRO 185, Hettich Lab Technology, Tuttlingen, Germany) at 10,000 rpm (1075 RFC) for 6 min. Subsequently, an aliquot of the filtrate obtained in each system was obtained and evaluated in a microplate reader (Synergy, H1. Microplate reader, Biotek, Winooski, VT, USA). The amount of ampicillin was determined by interpolation from a calibration curve that was previously prepared at the following concentrations using ultra-pure water as the solvent: 0.1, 0.2, 0.4, 0.6, 0.8, 1.0, and 1.2 mg/mL. The amount of ampicillin loaded inside the PECs was calculated using the following equation:

(1) $$EE=\frac{Q_{t}-Q_{s}}{Q_{t}}\times 100$$

where, *EE*, *Q_t_*, and *Q_s_* correspond to the encapsulation efficiency, initial total amount of ampicillin, and amount of ampicillin in the filtrate, respectively.

### 2.5. Antimicrobial Effect of Nanoparticles

The antimicrobial effect of antibiotic and ampicillin-loaded PECNs (including Blank-PECNs) was determined using the broth microdilution method according to the Clinical and Laboratory Standards Institute guidelines [80]. Briefly, *Staphylococcus aureus* ATCC25923, ATCC29213, and ATCC43300 were separately inoculated in a Mueller–Hinton broth (MHB) at 37 °C for 24 h and diluted with MHB broth until an optical density (absorbance at λ = 620 nm) of 0.1 (~1 × 10^8^ CFU/mL) was reached. Subsequently, a 1/1000 dilution factor was employed (~1 × 10^5^ UFC/mL), and 50 µL of this bacterial culture was incubated for 18–20 h in 96-well plates at 37 °C with 50 µL of selected nanoparticle samples that were sterilized through a 0.22 µm sterile nylon membrane. Two-fold serial dilutions that ranged from 0.008 to 256 µg/mL were used for each sample, and phosphate-buffered saline was used as a negative control. The minimal inhibitory concentration (MIC) was visually determined after incubation.

### 2.6. Statistical Analysis

Data were tabulated and analyzed using the Minitab^®^ v. 17 software (Minitab^®^ Inc., State College, PA, USA). The effects of the UHPH conditions in the particle size, PDI, and zeta potential were evaluated using the single-factor ANOVA test. The Dunnett post hoc test was utilized to determine significant differences between the independent groups and the control group (No UHPH). A confidence level of 95% was adopted, and data are expressed as the mean ± standard deviation.

## 3. Results and Discussion

### 3.1. Production and Characterization of Polyelectrolyte Complexes

**Figure 2:**
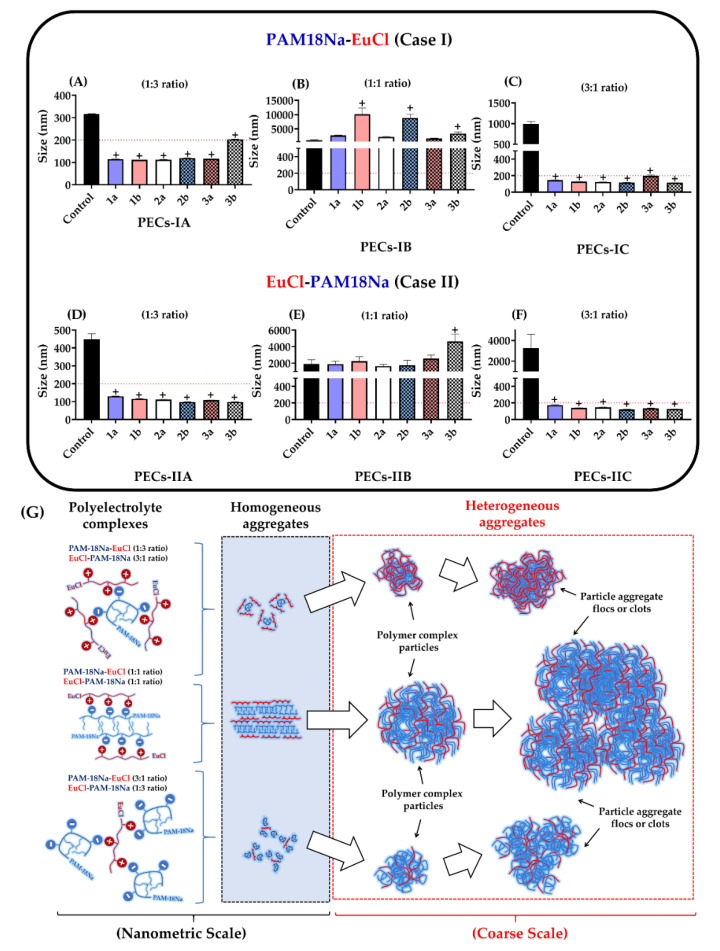
Characterization of particle size for ampicillin-loaded PECs. (**A**) PAM-18Na-EuCl 1:3 ratio. (**B**) PAM-18Na-EuCl 1:1 ratio. (**C**) PAM-18Na-EuCl 3:1 ratio. (**D**) EuCl-PAM-18Na 1:3 ratio. (**E**) EuCl-PAM-18Na 1:1 ratio. (**F**) EuCl-PAM-18Na 3:1 ratio. (**G**) scheme of the polyelectrolytic aggregates formation process in different size scales. The control corresponds to polyelectrolyte complexes obtained initially by polyelectrolytic complexation (bottom-up method) and was not subjected to UHPH (top-down method). “+” significant at *p* < 0.05.

**Figure 3:**
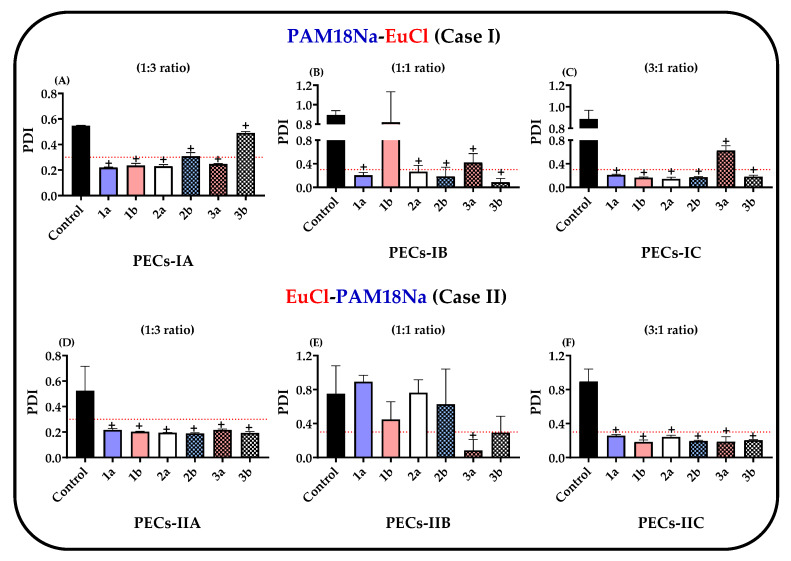
Characterization of the polydispersity index (PDI) for ampicillin-loaded PECs. (**A**) PAM-18Na-EuCl 1:3 ratio. (**B**) PAM-18Na-EuCl 1:1 ratio. (**C**) PAM-18Na-EuCl 3:1 ratio. (**D**) EuCl-PAM-18Na 1:3 ratio. (**E**) EuCl-PAM-18Na 1:1 ratio. (**F**) EuCl-PAM-18Na 3:1 ratio. The control corresponds to polyelectrolyte complexes obtained initially by polyelectrolytic complexation (bottom-up method) and was not subjected to UHPH (top-down method). “+” significant at *p* < 0.05.

**Figure 4:**
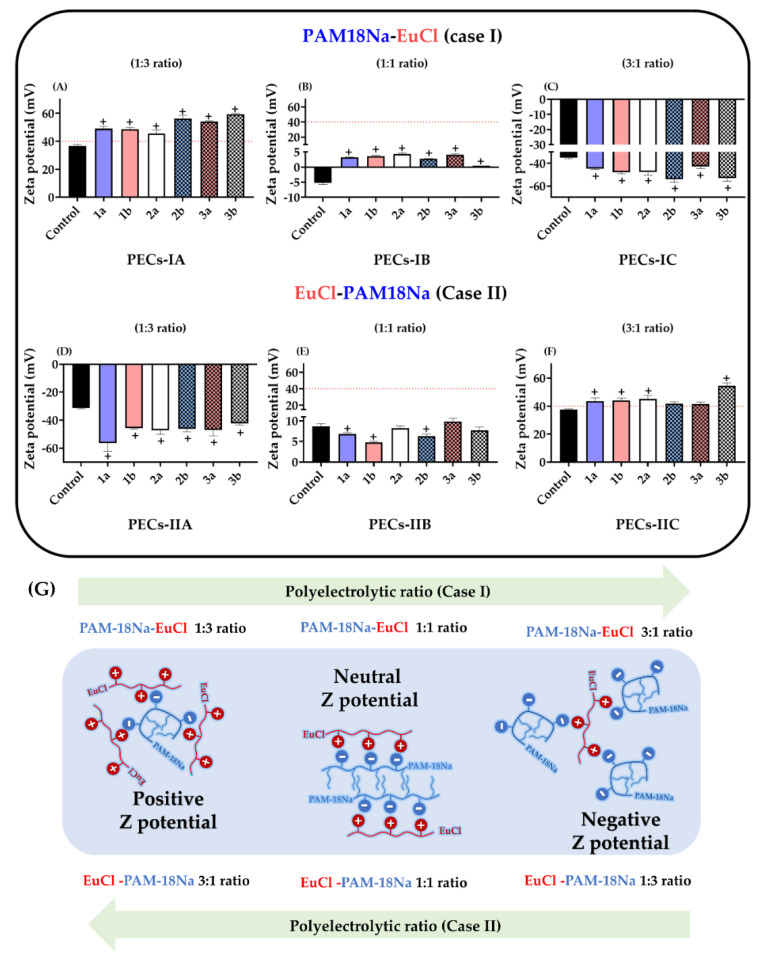
Characterization of zeta potential for ampicillin-loaded PECs. (**A**) PAM-18Na-EuCl 1:3 ratio. (**B**) PAM-18Na-EuCl 1:1 ratio. (**C**) PAM-18Na-EuCl 3:1 ratio. (**D**) EuCl-PAM-18Na 1:3 ratio. (**E**) EuCl-PAM-18Na 1:1 ratio. (**F**) EuCl-PAM-18Na 3:1 ratio. (**G**) Scheme of the formation of the PECs interfaces with different electrical polarization degrees. The control corresponds to polyelectrolyte complexes obtained initially by polyelectrolytic complexation (bottom-up method) and was not subjected to UHPH (top-down method). “+” significant at *p* < 0.05.

**Figure 5:**
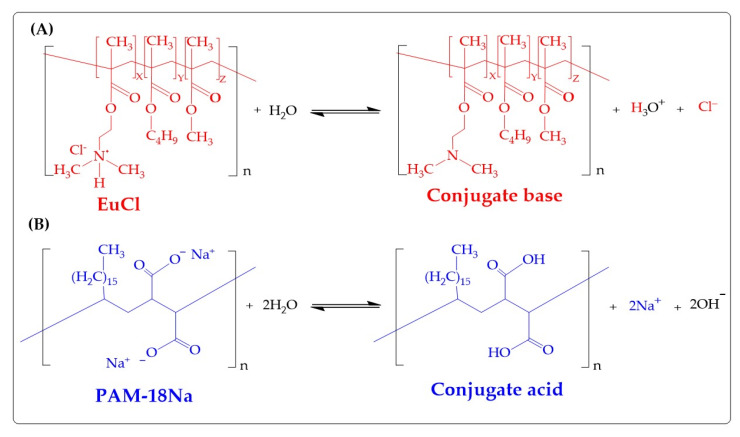
Chemical structure of the (**A**) PAM-18Na and (**B**) EuCl polyelectrolyte monomer units, as well as their ionization process in the aqueous medium.

The results of the physicochemical characterization of PECs (i.e., particle size, PDI, and zeta potential, pH and *EE*), obtained by polyelectrolyte complexation (bottom-up method) and subsequently by UHPH (top-down method), are presented in Figure 2, Figure 3, Figure 4 and Figure 5. Likewise, the physicochemical characterization data, as well as the statistical analysis for each PEC, are presented in the supplementary materials in Tables S1–S8. In order to provide an easier and harmonized discussion on the physicochemical characterizations of the forty-two developed polyelectrolytic complexes, the results were analyzed and discussed according to whether the UHPH led to statistically significant changes in the physicochemical parameters; i.e., if the top-down method caused significant changes in the polyelectrolyte complexes’ features previously obtained by the bottom-up method. Then, such characteristics were compared between the complexes that were analogous in quantity and composition but different in complexation order. Consequently, these analogue complexes [PAM-18Na-EuCl (3:1 ratio) and EuCl-PAM-18Na (1:3)], [PAM-18Na-EuCl (1:3 ratio) and EuCl-PAM-18Na (3:1)] and [PAM-18Na-EuCl (1:1 ratio) and EuCl-PAM-18Na (1:1)] were compared to each other and contrasted with several physicochemical quality criteria defined in this study (particle size < 200 nm, PDI < 0.3, zeta potential ~|40| mV and encapsulation efficiencies of ampicillin > 40%). 

#### 3.1.1. Particle Size

The results of the particle size of the PECs formed between PAM-18Na and EuCl, as well as their respective controls, are depicted in Figure 2A–F. The results showed that the polyelectrolyte complexes not subjected to UHPH and corresponding to the control described sizes > 200 nm in all cases, whereas for the PECs subjected to UHPH, their particle sizes fluctuated depending on the type of PECs, including those with similar composition and polymeric proportion. In the case of polyelectrolyte complexes obtained only by bottom-up method, that is, not subject to UHPH (controls), the particles sizes of the analogous complexes, PAM-18Na-EuCl (3:1 ratio) and EuCl-PAM-18Na (1:3 ratio), were 316.0 ± 2.0 nm and 3252.3 ± 1344.4 nm, respectively (Figure 2A,F). Additionally, the particles sizes of analogous complexes, PAM-18Na-EuCl (1:3 ratio) and EuCl-PAM-18Na (3:1 ratio), were 991.4.0 ± 2.0 nm and 449.0 ± 31.3 nm, respectively (Figure 2C,D). In contrast, the particles sizes of the analogous complexes, PAM-18Na-EuCl (1:1 ratio) and EuCl-PAM-18Na (1:1 ratio) described the largest particle sizes, with respective values of 1044.0 ± 167.0 nm and 1910.3 ± 517.9 nm (Figure 2B,C). These results suggest that there is a relationship between the polyelectrolytic proportion and the way that such complexes are formed. Broadly, the development of these PECs is mediated by electrostatic interactions of opposite charges between the polyelectrolytes [66,78,79,80]. Thus, the PECs with non-equivalent proportions (e.g., 1:3 or 3:1 ratios) could be created by compact and organized aggregates from few polymeric chains. On the contrary, PECs with equivalent proportions (e.g., 1:1 ratio) are generated by random aggregates, which in turn are produced by multiple entangled polymer chains that increase particle size as is depicted in Figure 2G. Likewise, it can be observed that the increase in the titrant polymer proportion (TP) regarding the receiving polymer (RP) entails an increase in particle size, regardless of the order of addition of the polyelectrolyte. This result can be explained considering that before the polyelectrolytic complexation takes place, the receiving polymer is dissolved in the aqueous medium, forming homogeneous interpolymer aggregates (Figure 2G). Thus, when the titrant polymer is added in low proportions (TP:RP 1:3 ratio), this is deposited on the receiver polymer aggregates, creating a new particle with a larger size. Then, the addition of a more titrant polymer (TP:RP ratio 1:1) causes a greater interaction between itself and the previously formed particle, producing a new heterogeneous polyelectrolytic complex of multiple entangled chains, increasing the particle size. Finally, the maximum evaluated addition of the titrant polymer (TP:RP 3:1 ratio) forms a new polyelectrolytic complex, where a new layer of polymeric aggregates is generated, conducting to a system with the largest particle size.

Furthermore, UHPH (top-down method) affected the particle size of PECs depending on the type and polyelectrolytic proportion. For instance, UHPH led to an increase in the particle size of the PAM-18Na-EuCl (1:1 ratio) complexes in all of the conditions that were evaluated (Figure 2B) and a slight increase in the particle size of the EuCl-PAM-18Na (1:1 ratio) complexes (Figure 2E). In contrast, UHPH led to a marked decrease (<200 nm) in the particle size of the complexes that were formed at different polyelectrolytic proportions (1:3 and 3:1 ratio). Therefore, these results suggest that PECs formed in equivalent proportions (1:1 ratio) could be formed by aggregates with high electrostatic cohesiveness, which are not easily disaggregated by cavitation and shear effects during UHPH [81,82,83]. However, the electrostatic interactions between both polyelectrolytes in the complexes with non-equivalent proportions (e.g., 1:3 and 3:1 ratios) are smaller and can be easily cleaved. This leads to multiple formation–deformation processes of polyelectrolyte aggregates until PECs with a uniform size are established.

#### 3.1.2. PDI

The PDI of the PECs and their respective controls are shown in Figure 3A–F and the results revealed that the PDI exhibited a similar behavior to those observed in particle size. 

Regarding the polyelectrolyte complexes obtained only by the bottom-up method and not subjected to UHPH (control), these showed that PDI values were > 0.3 in all cases, suggesting that the complexes are created by different numbers of polymer chains that lead to the formation of several PECs populations during the polyelectrolytic complexation. However, the polydispersity varied in complexes that were subjected to UHPH (top-down method) depending on the polyelectrolytic ratio employed. For instance, the PDI values fluctuated between 0.2 and 1.0 in complexes with equivalent proportions (e.g., 1:1 ratio) (Figure 2B,E) and on the contrary, the PDI values of complexes with non-equivalent proportions (e.g., 1:3 and 3:1 ratio) were decreased and reached values that were <0.3 in most cases. Therefore, these results confirm the hypothesis that complexes produced in equivalent proportions (e.g., 1:1 ratio) lead to the formation of aggregates with several polymer chains, which can be easily disaggregated throughout the homogenization process, causing multiple PECs with a wide distribution of particle sizes. Meanwhile, complexes with non-equivalent proportions (e.g., 1:3 and 3:1 ratios), lead to a controlled process of formation–deformation of polyelectrolytic aggregates until nanoparticles of polyelectrolyte complexes with a very uniform particle size are created.

#### 3.1.3. Zeta Potential

The results of the zeta potential for the polyelectrolyte complexes formed between PAM-18Na and EuCl, as well as their controls are presented in Figure 4A–F, where some differences were observed depending on the polyelectrolyte ratio employed and submission to the top-down method (UHPH). 

In the case of PECs obtained only by UHPH (control) and those with equivalent polymer ratios, the zeta potential values were low in magnitude and very close to zero (Figure 3B,E). This result was expected, because the 1:1 ratio used between both polyelectrolytes leads to the neutralization of polymeric charges between the anionic groups (carboxylate group) of the PAM-18Na and the cationic groups (dimethylammonium group) of EuCl, and this decreases the value of zeta potential. In contrast, the zeta potential values of PECs with non-equivalent proportions (e.g., 1:3 and 3:1 ratios) were > |40| mV in all cases, suggesting that such complexes keep a considerable surface charge fraction that provides them an electrostatic stabilization effect and avoids inter-PECs aggregation.

All these results are very consistent with the results of particle size and PDI. Specifically, the findings demonstrated that PECs with equivalent polyelectrolyte proportions (1:1 ratio) tended to form more cohesive systems, which were not affected by cavitation and shear during UHPH. Likewise, such complexes tend to produce poorly polarized surfaces, which leads to a random aggregation and the formation of multiple inter-PEC networks that increase the particle size and polydispersity. On the contrary, systems with non-equivalent polyelectrolytic proportions (e.g., 1:3 and 3:1 ratio) (Figure 3A,C,D,F) tend to form smaller cohesive complexes, which are formed and deformed during UHPH. In this way, the generation of highly polarized surfaces avoids the inter-PECs aggregation and leads to the formation of more compact and uniform macrostructures in size and polydispersity. It is also important to highlight the effect of the zeta potential inversion that was reached by the change in the proportion between the titrant and receiver polyelectrolytes (Figure 3G). In the case of the PAM-18Na-EuCl complexes, the zeta potential changed from positive to negative when the TP:RP ratio varied from 1:3 to 3:1. However, the EuCl-PAM-18Na complexes exhibited the opposite effect. Likewise, the zeta potential values of both compounds with equivalent polyelectrolyte proportions (e.g., 1:1 ratio) were very close to zero, indicating that the electrostatic neutralization point of the PECs is reached at this proportion.

A similar behavior was observed with respect to pH, as shown in Table 2, where depending on the type of case of polyelectrolytic complexation, the pH changes according to the proportion of polyelectrolyte in the aqueous medium.

Thus, in case 1, when the EuCl (cationic polyelectrolyte) acted as the receiver polymer, a pH value of 3.27 ± 0.01 was obtained, which is consistent because such polymer has an ammonium salt in its structure. Whereas in case 2, where the receiving polymer was the anionic polyelectrolyte PAM-18Na, the obtained pH value was 10.84 ± 0.01, which is also consistent, since this polymer has carboxylate groups in its structure (Figure 5). Consequently, when the polyelectrolytic complexation takes place, the pH of the medium changes, where in case 1, the system went from an acidic pH (3.27 ± 0.01) to a less acidic pH (4.85 ± 0.01), then to a slightly basic pH (8.75 ± 0.01) and finally, at a more basic pH (9.65 ± 0.01). While in case 2, the opposite effect was observed, where the system went from a very basic pH (10.84 ± 0.01) to a less basic pH (9.80 ± 0.01), then to a slightly basic pH (7.98 ± 0.01) and later at an acidic pH (4.70 ± 0.02). These results are very interesting because they show that during the formation of the PECs, depending on the ratio between the receiving and titrating polymer, the pH of the medium changes. Therefore, when the EuCl polyelectrolyte is in the aqueous medium, it passes from an ionic form (ammonium salt form) to a neutral form (dimethyl-amino form), releasing protons into the medium (leading to an acidic pH). On the contrary, when the PAM-18Na polyelectrolyte is in the medium, it goes from its ionic form (carboxylate form) to its neutral form (carboxylic acid), releasing hydroxyls to the medium (leading to a basic pH). In this way, when the polyelectrolytic complexation is carried out, the neutralization reaction is generated between the protons and the hydroxyl coming from the respective equilibria of the polyelectrolytes in solution, forming the polymeric complexes of opposite charge and the different pH values of the medium as is depicted in Figure 5.

### 3.2. Encapsulation Efficiency (EE)

**Figure 6:**
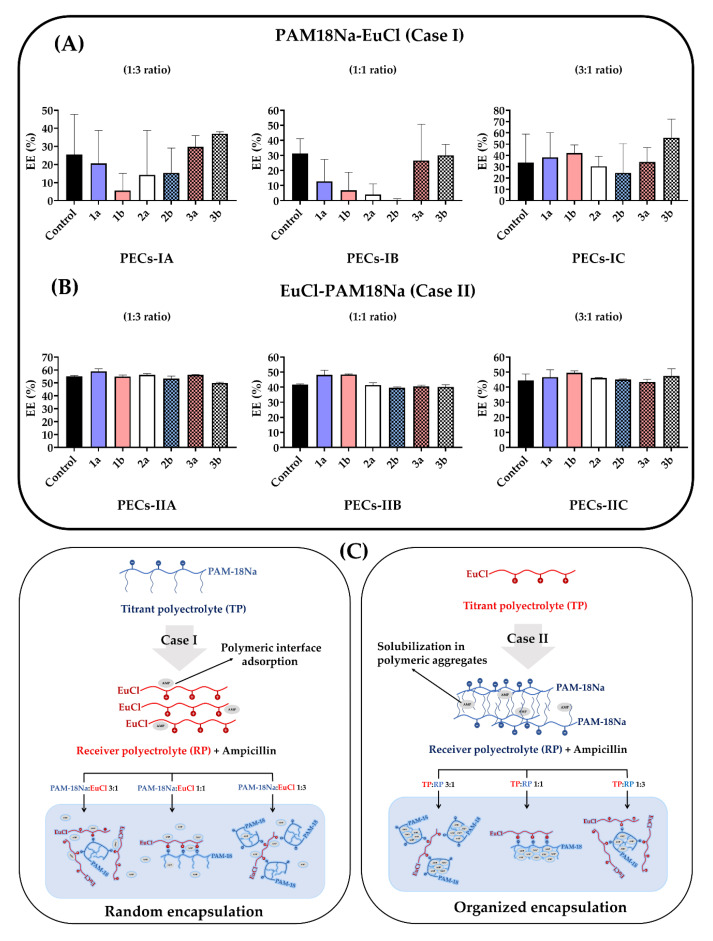
Ampicillin encapsulation efficiency in PECNs. (**A**) EuCl:PAM-18Na system. (**B**) PAM-18Na-EuCl system. (**C**) Schematic of the encapsulation process given during polyelectrolytic complexation. The control corresponds to the amount of ampicillin encapsulated in the polyelectrolyte complexes not subjected to UHPH.

The results of ampicillin encapsulation in the PECs are shown in Figure 6 and even though a normal distribution in *EE* data was reached, none of these fit to the ANOVA statistical model and therefore, it was not possible to establish a statistically significant comparison between treatments and control. Similarly, it is important to note that UHPH (bottom-up method) did not affect the ampicillin *EE*, while the type of PEC did. Furthermore, there was a lack of tendency and a fluctuation in the values of ampicillin encapsulation percentages of the PAM-18Na-EuCl complexes (Figure 6A). On the contrary, the EuCl-PAM-18Na complexes exhibited a uniform behavior, where the ampicillin *EE* was between 40% and 60% with a low standard deviation (Figure 6B). These results suggest that several mechanisms are involved in the ampicillin encapsulation process as is depicted in Figure 6C. To analyze this situation, it is necessary to consider the way in which ampicillin interacts with the receiving polyelectrolyte before polyelectrolytic complexation.

As previously described, in case I interactions between ampicillin and the receiver polyelectrolyte (EuCl) may be mediated by interfacial adsorption phenomena; this behavior was previously characterized by the authors in another research work [40]. Thus, when the polyelectrolytic complexation process begins, the ampicillin located at the polymeric interface is desorbed and replaced by the titrant polyelectrolyte (PAM-18Na), establishing a dynamic competition between the polyelectrolytes of the complex and the drug.

On the contrary, in case II (ampicillin with receiver polyelectrolyte PAM-18Na), the drug is located inside the hydrophobic aggregates that are formed by the side alkyl chains of the polymer [76]. Thus, when polyelectrolytic complexation takes place, EE is not affected and tends to remain invariable regardless of the conditions applied during UHPH.

Hence, the complexes that met the physicochemical quality criteria established in this study are presented in Table 3.

### 3.3. Antimicrobial Effect of PECNs

**Figure 7:**
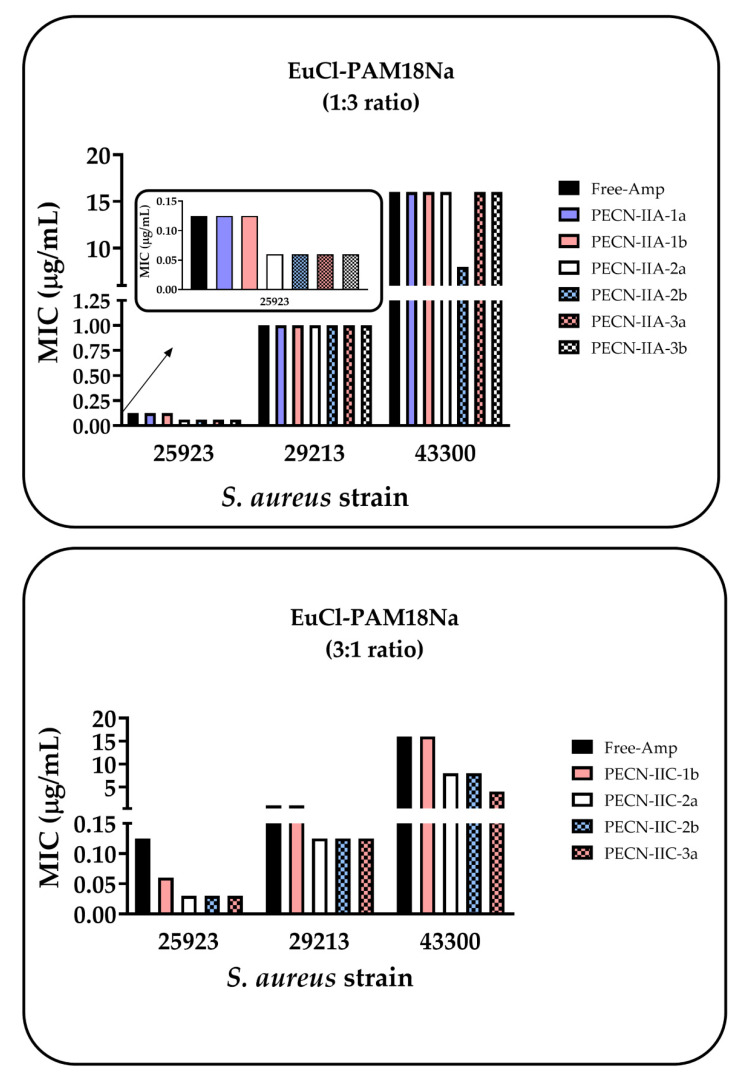
Antibacterial activity of the polyelectrolyte complexes that comply with the physicochemical quality criteria against *S. aureus* ATCC 25923, 29213, and 43300.

Antimicrobial activity assays for PECNs showed different behaviors depending on the family of the complex (Figure 7). Thus, no significant antimicrobial effect was appreciated in the case of the EuCl-PAM18Na (1:3 ratio) family, which was mainly composed of the anionic PAM18Na, since the MIC was very similar to that obtained with free ampicillin. Besides, a slight decrease in MIC with the ATCC 25923 strain (ampicillin sensitive) was observed in most of the PECNs (except for PECN-IIA-1b and 1b). However, this change was not important because this is a sensitive strain. In the case of the ATCC 29213 strain (resistant to ampicillin; sensitive to oxacillin), any change in MIC was observed with respect to free ampicillin and remained around 1 µg/mL. A similar result was observed with the ATCC43300 strain (resistant to methicillin), which has the highest resistance degree, and where the MIC remained invariable around 16 µg/mL, except with the PECN-IIA-1a complex, which presented a MIC of 8 µg/mL.

In contrast, the PAM-18Na-EuCl family, which was mainly composed of the cationic polymer, EuCl, slightly decreased the MIC of ampicillin for the three strains of *S. aureus*. In the case of the ATCC 25923 strain, a decrease in MIC was observed with most of the complexes in this family. In the case of the ATCC 29213 strain, there was a change in MIC with respect to free ampicillin from 1 to 0.125 µg/mL (except for PECN-IIC-1b). A similar result was observed with the ATCC43300 strain; there was a decrease from 16 to 8 µg/mL with PECN-IIC-2a and 2b, while PECN-IIC-3a decreased the MIC from 16 to 4 µg/mL. Although, there was a slight decrease in MIC with the PAM-18Na-EuCl family, these changes were not significant enough to be considered a promising antimicrobial effect. However, these results are interesting when they were compared to other types of nanosystems with similar characteristics that were evaluated using the same *S. aureus* strains. For instance, a study that utilized the PAM-18Na-ampicillin nanocomplexes showed that these NPs decreased the MIC by 75% in both resistant *S. aureus* strains [76]. Another study that used PECNs that were formed between chitosan and the anionic polyelectrolyte (PAM-18Na) showed that the MIC was reduced by 50% in both resistant strains [81]. Another study that used the EuCl cationic polyelectrolyte as a coating material on the surface of nanoliposomes described a marked decrease in MIC that was 4–18 times lower than that of free ampicillin [82]. On the other hand, previous studies have reported that free-Eudragit in solution exhibits toxic effects in human erythrocytes and cells due to a destabilizing effect on the membrane [83,84]. However, in another study, nanoparticle loading drugs were built with Eudragit and were safe and potentially offering enhancement in the pharmacological hepatoprotective properties [41]. 

The physicochemical properties of PECNs could influence antibacterial activity. According to the results obtained by z-potential (Figure 4), the anionic PAM18Na is located on the surface of the PECN for the EuCl-PAM18Na (1:3 ratio) family, and this polymer is constituted by carboxylate groups, which would have the ability to change the internal pH of the bacterial cell, leading to the interruption of the transport of the drug, either by changes in the permeability of the cell membrane or by the alteration of the proton flow mechanisms between the inside and the outside of the cell. [85]. In contrast, the PAM-18Na-EuCl family, where the complex surface consists mainly of the EuCl polymer (Figure 4), these could interact electrostatically with the bacterial surface, which is rich in anionic phospholipids such as phosphatidylglycerol [86], achieving a membrane-destabilizing effect and allowing the passage of water-soluble molecules [83]. On the other hand, the sizes exhibited by the PECNs (Figure 2) prevents them from entering through the pores of the bacteria cell wall since the latter have sizes between 2.03 and 3 nm [87]. Therefore, these results suggest that not only the physicochemical characteristics of the NPs, such as particle size, polydispersity, zeta potential, and EE, but also the composition of the nanoparticles influence the improvements in the antimicrobial effect.

## 4. Conclusions

It was found there was a relationship between the polyelectrolytic proportion and the way that such complexes are formed, where PECs with non-equivalent proportions are created by compact and organized aggregates from few polymeric chains, whilst the PECs with equivalent proportions are formed by random aggregates, which in turn are produced by multiple entangled polymer chains that increase particle size. Furthermore, UHPH (top-down method) affected the particle size and PDI of PECs depending on the type and polyelectrolytic proportion. In the case of PECs with equivalent proportions, the particle size and PDI considerably increase, while PEC with non-equivalent proportions reached size < 200 nm and PDI < 0.3. Regarding the zeta potential, it was found several differences depending on the polyelectrolyte ratio employed, where PECs with equivalent proportions described values very close to zero, while PECs with non-equivalent proportions showed high values around |40|. On the other hand, encapsulation of ampicillin showed a dependence on complexometric complexation and, specifically, on the receiving polymer. In the case of the EuCl cationic polymer, the ampicillin is adsorbed–desorbed in the polymer interface zone, leading to fluctuating and non-reproducible EE values. On the contrary, with the PAM-18Na polymer, ampicillin is contained within the polymeric side chains and therefore, the EE values were not affected, even when the PECs were subjected to UHPH. Regarding the results of the biological evaluation, it was found there was a slight decrease in MIC, but these changes were not significant enough to be considered a promising antimicrobial effect. Finally, it was proved that polyelectrolyte complexes formed between EuCl and PAM-18Na by means of polyelectrolytic complexation (bottom-up), led to systems with large particle sizes, high polydispersity and relatively low zeta potential values, while the submission of these systems to UHPH (top-down) caused a uniformity in such physicochemical characteristics, except in polyelectrolyte complexes with 1:1 ratio.

## Figures and Tables

**Table 1:** Experimental conditions employed in the development of polyelectrolyte complexes.

Complexation Conditions					UHPH Conditions					Polyelectrolyte Complex Name
Case I	Case II	Titrant: Receiver Ratio			Pressure (MPa)			Cycles		
PAM-18Na:EuCl	EuCl:PAM-18Na	A (1:3)	B (1:1)	C (3:1)	1 (69)	2 (138)	3 (172)	a (1)	b (4)	
x		x			No UHPH					(control)
x		x			x			x		PECs-IA-1a
x		x			x				x	PECs-IA-1b
x		x				x		x		PECs-IA-2a
x		x				x			x	PECs-IA-2b
x		x					x	x		PECs-IA-3a
x		x					x		x	PECs-IA-3b
x			x		No UHPH					(control)
x			x		x			x		PECs-IB-1a
x			x		x				x	PECs-IB-1b
x			x			x		x		PECs-IB-2a
x			x			x			x	PECs-IB-2b
x			x				x	x		PECs-IB-3a
x			x				x		x	PECs-IB-3b
x				x	No UHPH					(control)
x				x	x			x		PECs-IC-1a
x				x	x				x	PECs-IC-1b
x				x		x		x		PECs-IC-2a
x				x		x			x	PECs-IC-2b
x				x			x	x		PECs-IC-3a
x				x			x		x	PECs-IC-3b
	x	x			No UHPH					(control)
	x	x			x			x		PECs-IIA-1a
	x	x			x				x	PECs-IIA-1b
	x	x				x		x		PECs-IIA-2a
	x	x				x			x	PECs-IIA-2b
	x	x					x	x		PECs-IIA-3a
	x	x					x		x	PECs-IIA-3b
	x		x		No UHPH					(control)
	x		x		x			x		PECs-IIB-1a
	x		x		x				x	PECs-IB-1b
	x		x			x		x		PECs-IIB-2a
	x		x			x			x	PECs-IIB-2b
	x		x				x	x		PECs-IIB-3a
	x		x				x		x	PECs-IIB-3b
	x			x	No UHPH					(control)
	x			x	x			x		PECs-IIC-1a
	x			x	x				x	PECs-IIC-1b
	x			x		x		x		PEC-IIC-2a
	x			x		x			x	PEC-IIC-2b
	x			x			x	x		PEC-IIC-3a
	x			x			x		x	PEC-IIC-3b

**Table 2:** Change of the pH of the aqueous medium in the polyelectrolytic complexation process.

Case	System	Molar Ratio Regarding Monomer Units		pH
		Titrant Polymer	Receiver Polymers	
I	EuCl	0	1	3.27 ± 0.01
	PECNs-IA	1	3	4.85 ± 0.01
	PECNs-IB	1	1	8.75 ± 0.01
	PECNs-IC	3	1	9.65 ± 0.01
II	PAM-18Na	0	1	10.84 ± 0.01
	PECNs-IIA	1	3	9.80 ± 0.01
	PECNs-IIB	1	1	7.98 ± 0.01
	PECNs-IIC	3	1	4.70 ± 0.02

**Table 3:** Data of polyelectrolyte complexes that comply with the physicochemical quality criteria defined in the study (i.e., particle size < 200 nm, PDI < 0.3, zeta potential ~|40| mV, and encapsulation efficiencies of ampicillin > 40%).

PECNs Family	Polyelectrolyte Complex	Particle Size (nm)	PDI	Zeta Potential (mV)	Encapsulation Efficiency (%)
EuCl-PAM18Na (1:3 ratio)	PECN-IIA-1a	130.1 ± 1.4	0.217 ± 0.011	−56.6 ± 5.7	58.9 ± 2.0
	PECN-IIA-1b	115.8 ± 1.3	0.204 ± 0.003	−45.7 ± 0.7	54.8 ± 1.3
	PECN-IIA-2a	110.8 ± 1.1	0.194 ± 0.003	−47.4 ± 2.8	56.3 ± 1.1
	PECN-IIA-2b	98.0 ± 0.9	0.189 ± 0.007	−46.3 ± 2.1	53.2 ± 2.0
	PECN-IIA-3a	108.1 ± 0.9	0.215 ± 0.009	−47.0 ± 4.4	56.3 ± 0.3
	PECN-IIA-3b	97.5 ± 1.0	0.194 ± 0.012	−42.3 ± 1.3	50.0 ± 0.7
EuCl-PAM18Na (3:1 ratio)	PECN-IIC-1b	140.1 ± 2.1	0.185 ± 0.024	+43.9 ± 1.9	49.5 ± 1.4
	PECN-IIC-2a	145.8 ± 3.5	0.242 ± 0.022	+45.0 ± 2.6	46.1 ± 0.3
	PECN-IIC-2b	123.1 ± 0.9	0.194 ± 0.009	+41.7 ± 1.4	45.1 ± 0.5
	PECN-IIC-3a	135.4 ± 2.2	0.187 ± 0.058	+41.4 ± 1.6	43.5 ± 1.6

## Supplementary Materials

The following are available online at https://www.mdpi.com/2073-4360/12/5/1168/s1, Table S1: Physicochemical characterization of Blank polyelectrolyte complexes, Table S2: Statistical Dunnett analysis for particle size characterization of Blank PECs, Table S3: Statistical Dunnett analysis for Polydispersity Index characterization of Blank PECs, Table S4: Statistical Dunnett analysis for Zeta potential characterization of Blank PECs, Table S5: Physicochemical characterization of Ampicillin-loaded PECs, Table S6: Statistical Dunnett analysis for particle size characterization of Ampicillin-loaded PECs, Table S7: Statistical Dunnett analysis for Polydispersity Index characterization of Ampicillin-loaded PECs and Table S8: Statistical Dunnett analysis for Zeta potential characterization of Ampicillin-loaded PECs.
