# Supplementary material for: Development of Polyelectrolyte Complex Nanoparticles-PECNs Loaded with Ampicillin by Means of Polyelectrolyte Complexation and Ultra-High Pressure Homogenization (UHPH)
Source: Polymers (Basel). 2020 May 20;12(5):1168. doi: 10.3390/polym12051168 (PMC7285317; doi:10.3390/polym12051168)
Supplement: Supplementary file 1 [file polymers-12-01168-s001.docx]

**Table S1.** Physicochemical characterization of Blank polyelectrolyte complexes

| **Polyelectrolyte complex** | **Particle size (nm)** | **PDI** | **Zeta potential (mV)** |
| --- | --- | --- | --- |
| PECN-IA-0 | 240.3 ± 4.0 | 0.432 ± 0.026 | 33.3 ± 0.5 |
| PECN-IA-1a | 121.8 ± 1.0 | 0.208 ± 0.003 | 37.1 ± 2.1 |
| PECN-IA-1b | 115.6 ± 1.5 | 0.220 ± 0.011 | 40.4 ± 2.9 |
| PECN-IA-2a | 107.8 ± 0.4 | 0.201 ± 0.011 | 39.0 ± 2.8 |
| PECN-IA-2b | 99.9 ± 0.5 | 0.224 ± 0.018 | 38.6 ± 3.9 |
| PECN-IA-3a | 103.7 ± 0.4 | 0.218 ± 0.004 | 58.9 ± 0.9 |
| PECN-IA-3b | 101.1 ± 0.7 | 0.234 ± 0.004 | 62.2 ± 0.5 |
| PECN-IB-0 | 2397.3 ± 74.1 | 0.343 ± 0.130 | -32.0 ± 0.4 |
| PECN-IB-1a | 146.5 ± 0.8 | 0.224 ± 0.014 | -28.7 ± 1.9 |
| PECN-IB-1b | 123.7 ± 0.2 | 0.162 ± 0.004 | -50.7 ± 2.4 |
| PECN-IB-2a | 120.2 ± 0.4 | 0.183 ± 0.011 | -30.9 ± 1.9 |
| PECN-IB-2b | 108.1 ± 0.5 | 0.169 ± 0.008 | -52.6 ± 1.9 |
| PECN-IB-3a | 114.7 ± 0.8 | 0.187 ± 0.009 | -47.8 ± 3.1 |
| PECN-IB-3b | 105.7 ± 0.6 | 0.163 ± 0.004 | -53.4 ± 2.1 |
| PECN-IC-0 | 410.1 ± 11.2 | 0.557 ± 0.025 | -34.3 ± 1.5 |
| PECN-IC-1a | 120.5 ± 3.1 | 0.337 ± 0.006 | -54.8 ± 11.7 |
| PECN-IC-1b | 118.7 ± 2.0 | 0.355 ± 0.024 | -63.9 ± 6.4 |
| PECN-IC-2a | 109.3 ± 0.8 | 0.306 ± 0.036 | -47.8 ± 2.2 |
| PECN-IC-2b | 112.8 ± 0.4 | 0.380 ± 0.005 | -61.8 ± 7.5 |
| PECN-IC-3a | 104.6 ± 0.5 | 0.296 ± 0.028 | -48.3 ± 3.7 |
| PECN-IC-3b | 107.5 ± 0.3 | 0.319 ± 0.025 | -43.1 ± 3.0 |
| PECN-IIA-0 | 108.0 ± 1.1 | 0.473 ± 0.013 | -31.2 ± 2.9 |
| PECN-IIA-1a | 88.7 ± 3.2 | 0.457 ± 0.043 | -41.4 ± 2.5 |
| PECN-IIA-1b | 97.7 ± 8.1 | 0.434 ± 0.058 | -59.0 ± 1.5 |
| PECN-IIA-2a | 92.8 ± 0.8 | 0.471 ± 0.067 | -53.7 ± 2.1 |
| PECN-IIA-2b | 103.7 ± 2.2 | 0.455 ± 0.014 | -55.2 ± 2.5 |
| PECN-IIA-3a | 93.7 ± 2.7 | 0.508 ± 0.072 | -55.7 ± 3.4 |
| PECN-IIA-3b | 108.6 ± 4.8 | 0.435 ± 0.026 | -59.1 ± 3.5 |
| PECN-IIB-0 | 280.6 ± 2.7 | 0.489 ± 0.017 | -30.6 ± 1.8 |
| PECN-IIB-1a | 115.6 ± 0.8 | 0.194 ± 0.012 | -36.7 ± 3.3 |
| PECN-IIB-1b | 105.2 ± 1.9 | 0.184 ± 0.010 | -44.7 ± 2.5 |
| PECN-IIB-2a | 100.8 ± 1.4 | 0.210 ± 0.015 | -40.0 ± 2.7 |
| PECN-IIB-2b | 109.4 ± 1.4 | 0.303 ± 0.008 | -37.0 ± 3.2 |
| PECN-IIB-3a | 105.7 ± 2.5 | 0.287 ± 0.032 | -34.6 ± 4.0 |
| PECN-IIB-3b | 89.8 ± 0.6 | 0.215 ± 0.004 | -46.9 ± 2.5 |
| PECNs-IIC-0 | 1394.3 ± 136.0 | 0.928 ± 0.087 | 35.4 ± 0.4 |
| PECNs-IIC-1a | 142.0 ± 0.7 | 0.234 ± 0.002 | 49.0 ± 2.5 |
| PECNs-IIC-1b | 139.9 ± 3.6 | 0.256 ± 0.007 | 53.0 ± 5.3 |
| PECNs-IIC-2a | 121.2 ± 1.1 | 0.264 ± 0.014 | 48.2 ± 0.8 |
| PECNs-IIC-2b | 112.6 ± 1.7 | 0.249 ± 0.011 | 51.0 ± 2.9 |
| PECNs-IIC-3a | 113.8 ± 0.7 | 0.229 ± 0.010 | 45.2 ± 3.0 |
| PECNs-IIC-3b | 103.2 ± 1.6 | 0.214 ± 0.012 | 44.9 ± 1.0 |

**Table S2.** Statistical Dunnett analysis for particle size characterization of Blank PECs

| **Polyelectrolyte complex** | **Average size (nm)** | **Group** |
| --- | --- | --- |
| PECN-IA-0 (control) | 240.3 | A |
| PECN-IA-1a | 121.8 |  |
| PECN-IA-1b | 115.6 |  |
| PECN-IA-2a | 107.8 |  |
| PECN-IA-3a | 103.7 |  |
| PECN-IA-3b | 101.1 |  |
| PECN-IA-2b | 99.9 |  |
| **Polyelectrolyte complex** | **Average size (nm)** | **Group** |
| PECN-IB-0 (control) | 2397.3 | A |
| PECN-IB-1a | 146.5 |  |
| PECN-IB-1b | 123.7 |  |
| PECN-IB-2a | 120.2 |  |
| PECN-IB-3a | 114.7 |  |
| PECN-IB-2b | 108.1 |  |
| PECN-IB-3b | 105.7 |  |
| **Polyelectrolyte complex** | **Average size (nm)** | **Group** |
| PECN-IC-0 (control) | 410.1 | A |
| PECN-IC-1a | 120.5 |  |
| PECN-IC-1b | 118.7 |  |
| PECN-IC-2b | 112.8 |  |
| PECN-IC-2a | 109.3 |  |
| PECN-IC-3b | 107.5 |  |
| PECN-IC-3a | 104.6 |  |
| **Polyelectrolyte complex** | **Average size (nm)** | **Group** |
| PECN-IIA-0 (control) | 108.0 | A |
| PECN-IIA-3b | 108.6 | A |
| PECN-IIA-2b | 103.7 | A |
| PECN-IIA-1b | 97.7 |  |
| PECN-IIA-3a | 93.7 |  |
| PECN-IIA-2a | 92.8 |  |
| PECN-IIA-1a | 88.8 |  |
| **Polyelectrolyte complex** | **Average size (nm)** | **Group** |
| PECN-IIB-0 (control) | 280.6 | A |
| PECN-IIB-1a | 115.6 |  |
| PECN-IIB-2b | 109.4 |  |
| PECN-IIB-3a | 105.7 |  |
| PECN-IIB-1b | 105.2 |  |
| PECN-IIB-2a | 100.8 |  |
| PECN-IIB-3b | 89.8 |  |
| **Polyelectrolyte complex** | **Average size (nm)** | **Group** |
| PECN-IIC-0 (control) | 1394.3 | A |
| PECN-IIC-1a | 142.0 |  |
| PECN-IIC-1b | 139.9 |  |
| PECN-IIC-2a | 121.2 |  |
| PECN-IIC-3a | 113.8 |  |
| PECN-IIC-2b | 112.6 |  |
| PECN-IIC-3b | 103.2 |  |

**Table S3**. Statistical Dunnett analysis for Polydispersity Index characterization of Blank PECs.

| **Polyelectrolyte complex** | **Average PDI** | **Group** |
| --- | --- | --- |
| PECN-IA-0 (control) | 0.432 | A |
| PECN-IA-3b | 0.234 |  |
| PECN-IA-2b | 0.224 |  |
| PECN-IA-1b | 0.220 |  |
| PECN-IA-3a | 0.218 |  |
| PECN-IA-1a | 0.208 |  |
| PECN-IA-2a | 0.201 |  |
| **Polyelectrolyte complex** | **Average PDI** | **Group** |
| PECN-IB-0 (control) | 0.343 | A |
| PECN-IB-1a | 0.224 |  |
| PECN-IB-3a | 0.187 |  |
| PECN-IB-2a | 0.183 |  |
| PECN-IB-2b | 0.169 |  |
| PECN-IB-3b | 0.163 |  |
| PECN-IB-1b | 0.162 |  |
| **Polyelectrolyte complex** | **Average PDI** | **Group** |
| PECN-IC-0 (control) | 0.557 | A |
| PECN-IC-2b | 0.380 |  |
| PECN-IC-1b | 0.355 |  |
| PECN-IC-1a | 0.337 |  |
| PECN-IC-3b | 0.319 |  |
| PECN-IC-2a | 0.306 |  |
| PECN-IC-3a | 0.296 |  |
| **Polyelectrolyte complex** | **Average PDI** | **Group** |
| PECN-IIA-0 (control) | 0.473 | A |
| PECN-IIA-3a | 0.508 | A |
| PECN-IIA-2a | 0.471 | A |
| PECN-IIA-1a | 0.457 | A |
| PECN-IIA-2b | 0.455 | A |
| PECN-IIA-3b | 0.435 | A |
| PECN-IIA-1b | 0.434 | A |
| **Polyelectrolyte complex** | **Average PDI** | **Group** |
| PECN-IIB-0 (control) | 0.489 | A |
| PECN-IIB-2b | 0.303 |  |
| PECN-IIB-3a | 0.287 |  |
| PECN-IIB-3b | 0.215 |  |
| PECN-IIB-2a | 0.210 |  |
| PECN-IIB-1a | 0.194 |  |
| PECN-IIB-1b | 0.184 |  |
| **Polyelectrolyte complex** | **Average PDI** | **Group** |
| PECN-IIC-0 (control) | 0.928 | A |
| PECN-IIC-2a | 0.264 |  |
| PECN-IIC-1b | 0.256 |  |
| PECN-IIC-2b | 0.249 |  |
| PECN-IIC-1a | 0.234 |  |
| PECN-IIC-3a | 0.229 |  |
| PECN-IIC-3b | 0.214 |  |

**Table S4.** Statistical Dunnett analysis for Zeta potential characterization of Blank PECs

| **Polyelectrolyte complex** | **Average Zeta potential (mV)** | **Group** |
| --- | --- | --- |
| PECN-IA-0 (control) | 33.3 | A |
| PECN-IA-3b | 62.2 |  |
| PECN-IA-3a | 58.9 |  |
| PECN-IA-1b | 40.4 |  |
| PECN-IA-2a | 39.0 |  |
| PECN-IA-2b | 38.6 | A |
| PECN-IA-1a | 37.1 | A |
| **Polyelectrolyte complex** | **Average Zeta potential (mV)** | **Group** |
| PECN-IB-0 (control) | -32.0 | A |
| PECN-IB-1a | -28.7 | A |
| PECN-IB-2a | -30.9 | A |
| PECN-IB-3a | -47.8 |  |
| PECN-IB-1b | -50.7 |  |
| PECN-IB-2b | -52.6 |  |
| PECN-IB-3b | -53.4 |  |
| **Polyelectrolyte complex** | **Average Zeta potential (mV)** | **Group** |
| PECN-IC-0 (control) | -34.3 | A |
| PECN-IC-3b | -43.1 | A |
| PECN-IC-2a | -47.8 | A |
| PECN-IC-3a | -48.3 | A |
| PECN-IC-1a | -54.8 |  |
| PECN-IC-2b | -61.8 |  |
| PECN-IC-1b | -63.9 |  |
| **Polyelectrolyte complex** | **Average Zeta potential (mV)** | **Group** |
| PECN-IIA-0 (control) | -31.2 | A |
| PECN-IIA-1a | -41.4 |  |
| PECN-IIA-2a | -53.7 |  |
| PECN-IIA-2b | -55.2 |  |
| PECN-IIA-3a | -55.7 |  |
| PECN-IIA-1b | -59.0 |  |
| PECN-IIA-3b | -59.1 |  |
| **Polyelectrolyte complex** | **Average Zeta potential (mV)** | **Group** |
| PECN-IIB-0 (control) | -30.6 | A |
| PECN-IIB-3a | -34.6 | A |
| PECN-IIB-1a | -36.7 | A |
| PECN-IIB-2b | -37.0 | A |
| PECN-IIB-2a | -40.0 |  |
| PECN-IIB-1b | -44.7 |  |
| PECN-IIB-3b | -46.9 |  |
| **Polyelectrolyte complex** | **Average Zeta potential (mV)** | **Group** |
| PECN-IIC-0 (control) | 35.4 | A |
| PECN-IIC-1b | 53.0 |  |
| PECN-IIC-2b | 51.0 |  |
| PECN-IIC-1a | 49.0 |  |
| PECN-IIC-2a | 48.2 |  |
| PECN-IIC-3a | 45.2 |  |
| PECN-IIC-3b | 44.9 |  |

**Table S5.** Physicochemical characterization of Ampicillin-loaded PECs

| **Polyelectrolyte complex** | **Particle size (nm)** | **PDI** | **Zeta potential (mV)** | **Encapsulation efficiency (%)** |
| --- | --- | --- | --- | --- |
| PECN-IA-0 | 316.0 ± 2.0 | 0.546 ± 0.005 | 36.7 ± 1.1 | 25.6 ± 22.2 |
| PECN-IA-1a | 114.3 ± 0.6 | 0.220 ± 0.005 | 49.0 ± 1.7 | 20.6 ± 18.3 |
| PECN-IA-1b | 111.7 ± 0.3 | 0.235 ± 0.017 | 48.6 ± 1.5 | 5.6 ± 9.6 |
| PECN-IA-2a | 111.9 ± 0.9 | 0.229 ± 0.014 | 45.5 ± 2.7 | 14.2 ± 24.6 |
| PECN-IA-2b | 119.2 ± 0.6 | 0.307 ± 0.029 | 56.2 ± 2.4 | 15.3 ± 13.7 |
| PECN-IA-3a | 116.0 ± 0.5 | 0.247 ± 0.004 | 54.0 ± 0.7 | 29.7 ± 6.2 |
| PECN-IA-3b | 203.4 ± 0.8 | 0.491 ± 0.011 | 59.3 ± 0.9 | 37.0 ± 1.1 |
| PECN-IB-0 | 1044.0 ± 167.0 | 0.894 ± 0.045 | -5.2 ± 0.5 | 31.2 ± 9.9 |
| PECN-IB-1a | 2700.3 ± 107.4 | 0.203 ± 0.051 | 3.2 ± 0.1 | 12.8 ± 14.5 |
| PECN-IB-1b | 10096.0 ± 2166.0 | 0.819 ± 0.314 | 3.6 ± 0.3 | 6.9 ± 12.0 |
| PECN-IB-2a | 2204.3 ± 159.1 | 0.264 ± 0.106 | 4.3 ± 0.3 | 4.0 ± 7.0 |
| PECN-IB-2b | 8825.3 ± 1322.6 | 0.185 ± 0.156 | 2.8 ± 0.1 | 0.5 ± 0.9 |
| PECN-IB-3a | 1576.3 ± 159.3 | 0.419 ± 0.152 | 4.1 ± 0.3 | 26.6 ± 24.0 |
| PECN-IB-3b | 3395.3 ± 517.0 | 0.084 ± 0.067 | 0.0 ± 0.0 | 30.1 ± 7.3 |
| PECN-IC-0 | 991.4 ± 55.4 | 0.885 ± 0.083 | -35.1 ± 0.7 | 33.6 ± 25.2 |
| PECN-IC-1a | 146.8 ± 2.1 | 0.210 ± 0.014 | -44.7 ± 0.9 | 38.2 ± 21.9 |
| PECN-IC-1b | 128.9 ± 0.8 | 0.164 ± 0.013 | -48.3 ± 1.8 | 42.0 ± 7.3 |
| PECN-IC-2a | 124.1 ± 1.0 | 0.143 ± 0.030 | -47.7 ± 2.8 | 30.4 ± 8.9 |
| PECN-IC-2b | 116.4 ± 2.4 | 0.173 ± 0.013 | -54.0 ± 2.7 | 24.5 ± 25.9 |
| PECN-IC-3a | 199.5 ± 11.1 | 0.622 ± 0.083 | -42.9 ± 1.6 | 34.3 ± 12.7 |
| PECN-IC-3b | 115.8 ± 0.4 | 0.181 ± 0.025 | -53.0 ± 2.8 | 55.7 ± 16.3 |
| PECN-IIA-0 | 449.0 ± 31.3 | 0.523 ± 0.192 | -31.5 ± 0.4 | 55.2 ± 0.7 |
| PECN-IIA-1a | 130.1 ± 1.4 | 0.217 ± 0.011 | -56.6 ± 5.7 | 58.9 ± 2.0 |
| PECN-IIA-1b | 115.8 ± 1.3 | 0.204 ± 0.003 | -45.7 ± 0.7 | 54.8 ± 1.3 |
| PECN-IIA-2a | 110.8 ± 1.1 | 0.194 ± 0.003 | -47.4 ± 2.8 | 56.3 ± 1.1 |
| PECN-IIA-2b | 98.0 ± 0.9 | 0.189 ± 0.007 | -46.3 ± 2.1 | 53.2 ± 2.0 |
| PECN-IIA-3a | 108.1 ± 0.9 | 0.215± 0.009 | -47.0 ± 4.4 | 56.3 ± 0.3 |
| PECN-IIA-3b | 97.5 ± 1.0 | 0.194 ± 0.012 | -42.3 ± 1.3 | 50.0 ± 0.7 |
| PECN-IIB-0 | 1910.3 ± 517.9 | 0.749 ± 0.330 | 8.7 ± 0.7 | 41.7 ± 0.4 |
| PECN-IIB-1a | 1899.7 ± 344.0 | 0.893 ± 0.075 | 6.8 ± 0.4 | 48.2 ± 3.1 |
| PECN-IIB-1b | 2260.0 ± 515.3 | 0.449 ± 0.208 | 4.8 ± 0.3 | 48.4 ± 0.4 |
| PECN-IIB-2a | 1639.3 ± 219.1 | 0.763 ± 0.151 | 8.2 ± 0.5 | 41.4 ± 1.6 |
| PECN-IIB-2b | 1754.3 ± 607.4 | 0.626 ± 0.413 | 6.3 ± 0.6 | 39.6 ± 0.6 |
| PECN-IIB-3a | 2568.3± 420.1 | 0.083 ± 0.130 | 9.9 ± 0.8 | 40.6 ± 0.8 |
| PECN-IIB-3b | 4623.7 ± 889.9 | 0.293 ± 0.194 | 7.7± 0.9 | 40.2 ± 1.5 |
| PECNs-IIC-0 | 3252.3 ± 1344.4 | 0.896 ± 0.145 | 37.4 ± 0.6 | 44.6 ± 4.1 |
| PECNs-IIC-1a | 174.7 ± 5.1 | 0.256 ± 0.016 | 43.5 ± 2.5 | 46.7 ± 5.0 |
| PECNs-IIC-1b | 140.1 ± 2.1 | 0.185 ± 0.024 | 43.9 ± 1.9 | 49.5 ± 1.4 |
| PECNs-IIC-2a | 145.8 ± 3.5 | 0.242 ± 0.022 | 45.0 ± 2.6 | 46.1 ± 0.3 |
| PECNs-IIC-2b | 123.1 ± 0.9 | 0.194 ± 0.009 | 41.7 ± 1.4 | 45.1 ± 0.5 |
| PECNs-IIC-3a | 135.4 ± 2.2 | 0.187 ± 0.058 | 41.4 ± 1.6 | 43.5 ± 1.6 |
| PECNs-IIC-3b | 125.3 ± 1.3 | 0.204 ± 0.010 | 54.5 ± 2.1 | 47.4 ± 4.7 |

**Table S6.** Statistical Dunnett analysis for particle size characterization of Ampicillin-loaded PECs

| **Polyelectrolyte complex** | **Average size (nm)** | **Group** |
| --- | --- | --- |
| PECN-IA-0 (control) | 316.0 | A |
| PECN-IA-3b | 203.4 |  |
| PECN-IA-2b | 119.2 |  |
| PECN-IA-3a | 116.0 |  |
| PECN-IA-1a | 114.3 |  |
| PECN-IA-2a | 111.9 |  |
| PECN-IA-1b | 111.7 |  |
| **Polyelectrolyte complex** | **Average size (nm)** | **Group** |
| PECN-IB-0 (control) | 1044.0 | A |
| PECN-IB-1b | 10096.0 |  |
| PECN-IB-2b | 8825.0 |  |
| PECN-IB-3b | 3395.0 |  |
| PECN-IB-1a | 2700.3 | A |
| PECN-IB-2a | 2204.3 | A |
| PECN-IB-3a | 1576.3 | A |
| **Polyelectrolyte complex** | **Average size (nm)** | **Group** |
| PECN-IC-0 (control) | 991.4 | A |
| PECN-IC-3a | 199.5 |  |
| PECN-IC-1a | 146.8 |  |
| PECN-IC-1b | 128.9 |  |
| PECN-IC-2a | 124.1 |  |
| PECN-IC-2b | 116.4 |  |
| PECN-IC-3b | 115.8 |  |
| **Polyelectrolyte complex** | **Average size (nm)** | **Group** |
| PECN-IIA-0 (control) | 449.0 | A |
| PECN-IIA-1a | 130.1 |  |
| PECN-IIA-1b | 115.8 |  |
| PECN-IIA-2a | 110.8 |  |
| PECN-IIA-3a | 108.1 |  |
| PECN-IIA-2b | 98.0 |  |
| PECN-IIA-3b | 97.5 |  |
| **Polyelectrolyte complex** | **Average size (nm)** | **Group** |
| PECN-IIB-0 (control) | 1910.0 | A |
| PECN-IIB-3b | 4624.0 |  |
| PECN-IIB-3a | 2568.0 | A |
| PECN-IIB-1b | 2260.0 | A |
| PECN-IIB-1a | 1900.0 | A |
| PECN-IIB-2b | 1754.0 | A |
| PECN-IIB-2a | 1639.0 | A |
| **Polyelectrolyte complex** | **Average size (nm)** | **Group** |
| PECN-IIC-0 (control) | 3252.0 | A |
| PECN-IIC-1a | 174.7 |  |
| PECN-IIC-2a | 145.8 |  |
| PECN-IIC-1b | 140.1 |  |
| PECN-IIC-3a | 135.4 |  |
| PECN-IIC-3b | 125.3 |  |
| PECN-IIC-2b | 123.1 |  |

**Table S7**. Statistical Dunnett analysis for Polydispersity Index characterization of Ampicillin-loaded PECs

| **Polyelectrolyte complex** | **Average PDI** | **Group** |
| --- | --- | --- |
| PECN-IA-0 (control) | 0.546 | A |
| PECN-IA-3b | 0.491 |  |
| PECN-IA-2b | 0.307 |  |
| PECN-IA-3a | 0.247 |  |
| PECN-IA-1b | 0.235 |  |
| PECN-IA-2a | 0.229 |  |
| PECN-IA-1a | 0.220 |  |
| **Polyelectrolyte complex** | **Average PDI** | **Group** |
| PECN-IB-0 (control) | 0.894 | A |
| PECN-IB-1b | 0.819 | A |
| PECN-IB-3a | 0.419 |  |
| PECN-IB-2a | 0.264 |  |
| PECN-IB-1a | 0.203 |  |
| PECN-IB-2b | 0.185 |  |
| PECN-IB-3b | 0.084 |  |
| **Polyelectrolyte complex** | **Average PDI** | **Group** |
| PECN-IC-0 (control) | 0.885 | A |
| PECN-IC-3a | 0.622 |  |
| PECN-IC-1a | 0.2103 |  |
| PECN-IC-3b | 0.181 |  |
| PECN-IC-2b | 0.1727 |  |
| PECN-IC-1b | 0.1637 |  |
| PECN-IC-2a | 0.143 |  |
| **Polyelectrolyte complex** | **Average PDI** | **Group** |
| PECN-IIA-0 (control) | 0.523 | A |
| PECN-IIA-1a | 0.217 |  |
| PECN-IIA-3a | 0.215 |  |
| PECN-IIA-1b | 0.204 |  |
| PECN-IIA-2a | 0.194 |  |
| PECN-IIA-3b | 0.194 |  |
| PECN-IIA-2b | 0.189 |  |
| **Polyelectrolyte complex** | **Average PDI** | **Group** |
| PECN-IIB-0 (control) | 0.749 | A |
| PECN-IIB-1a | 0.893 | A |
| PECN-IIB-2a | 0.763 | A |
| PECN-IIB-2b | 0.626 | A |
| PECN-IIB-1b | 0.449 | A |
| PECN-IIB-3b | 0.293 | A |
| PECN-IIB-3a | 0.083 |  |
| **Polyelectrolyte complex** | **Average PDI** | **Group** |
| PECN-IIC-0 (control) | 0.896 | A |
| PECN-IIC-1a | 0.256 |  |
| PECN-IIC-2a | 0.242 |  |
| PECN-IIC-3b | 0.204 |  |
| PECN-IIC-2b | 0.194 |  |
| PECN-IIC-3a | 0.187 |  |
| PECN-IIC-1b | 0.185 |  |

**Table S8.** Statistical Dunnett analysis for Zeta potential characterization of Ampicillin-loaded PECs

| **Polyelectrolyte complex** | **Average Zeta potential (mV)** | **Group** |
| --- | --- | --- |
| PECN-IA-0 (control) | 36.7 | A |
| PECN-IA-3b | 59.3 |  |
| PECN-IA-2b | 56.2 |  |
| PECN-IA-3a | 54.0 |  |
| PECN-IA-1a | 49.0 |  |
| PECN-IA-1b | 48.6 |  |
| PECN-IA-2a | 45.5 |  |
| **Polyelectrolyte complex** | **Average Zeta potential (mV)** | **Group** |
| PECN-IB-0 (control) | -5.2 | A |
| PECN-IB-2a | 4.3 |  |
| PECN-IB-3a | 4.1 |  |
| PECN-IB-1b | 3.6 |  |
| PECN-IB-1a | 3.2 |  |
| PECN-IB-2b | 2.8 |  |
| PECN-IB-3b | -0.01 |  |
| **Polyelectrolyte complex** | **Average Zeta potential (mV)** | **Group** |
| PECN-IC-0 (control) | -35.1 | A |
| PECN-IC-3a | -42.9 |  |
| PECN-IC-1a | -44.7 |  |
| PECN-IC-2a | -47.7 |  |
| PECN-IC-1b | -47.8 |  |
| PECN-IC-3b | -53.0 |  |
| PECN-IC-2b | -54.0 |  |
| **Polyelectrolyte complex** | **Average Zeta potential (mV)** | **Group** |
| PECN-IIA-0 (control) | -31.5 | A |
| PECN-IIA-3b | -42.3 |  |
| PECN-IIA-1b | -45.7 |  |
| PECN-IIA-2b | -46.3 |  |
| PECN-IIA-3a | -47.0 |  |
| PECN-IIA-2a | -47.4 |  |
| PECN-IIA-1a | -56.6 |  |
| **Polyelectrolyte complex** | **Average Zeta potential (mV)** | **Group** |
| PECN-IIB-0 (control) | 8.7 | A |
| PECN-IIB-3a | 9.9 | A |
| PECN-IIB-2a | 8.2 | A |
| PECN-IIB-3b | 7.7 | A |
| PECN-IIB-1a | 6.8 |  |
| PECN-IIB-2b | 6.3 |  |
| PECN-IIB-1b | 4.8 |  |
| **Polyelectrolyte complex** | **Average Zeta potential (mV)** | **Group** |
| PECN-IIC-0 (control) | 37.4 | A |
| PECN-IIC-3b | 54.5 |  |
| PECN-IIC-2a | 45.0 |  |
| PECN-IIC-1b | 43.9 |  |
| PECN-IIC-1a | 43.5 |  |
| PECN-IIC-2b | 41.7 | A |
| PECN-IIC-3a | 41.4 | A |
